# A Novel Acetylation-Immune Subtyping for the Identification of a BET Inhibitor-Sensitive Subgroup in Melanoma

**DOI:** 10.3390/ph16071037

**Published:** 2023-07-21

**Authors:** Liuying Wang, Liuchao Zhang, Shuang Li, Lei Cao, Kang Li, Weiwei Zhao

**Affiliations:** 1 Department of Biostatistics, School of Public Health, Harbin Medical University, Harbin 150000, China; wangliuying@hrbmu.edu.cn (L.W.); zhangliu@hrbmu.edu.cn (L.Z.); 202101035@hrbmu.edu.cn (S.L.); caolei@hrbmu.edu.cn (L.C.); 2 MSD R&D (China) Co., Ltd., Beijing 100012, China

**Keywords:** histone acetylation, melanoma, acetylation-immune subtype, BET inhibitors, JQ-1, *KAT2A*, *HAT1*, immunotherapy

## Abstract

**Simple Summary:**

BET inhibitors (BETis) are a class of promising therapies that inhibit the growth of melanoma cells. However, how to identify BETi-sensitive subtypes of melanoma is still unclear. We analyzed 48 melanoma cell lines and 104 melanoma patients and identified two acetylation-immune subtypes (ALISs) in the cell lines and three ALISs in the patients. The ALIS I cell lines showed a higher sensitivity to the BET inhibitor JQ-1, while the ALIS I patients had the poorest survival outcomes. We also compared immune-related gene sets and key mRNAs associated with histone acetylation and JQ-1 sensitivity among the three ALISs. The results indicated that the sensitivity of the ALIS I cell lines to JQ-1 might be attributable to the impact of the low expression of *KAT2A* and the high expression of *HAT1* on the immune microenvironment. The ALIS I patients demonstrated sensitivity to BET inhibitors and B-cell-related immunotherapy, but did not respond to BRAF inhibitors.

**Abstract:**

Background: There have been significant advancements in melanoma therapies. BET inhibitors (BETis) show promise in impairing melanoma growth. However, identifying BETi-sensitive melanoma subtypes is challenging. Methods and Results: We analyzed 48 melanoma cell lines and 104 patients and identified two acetylation-immune subtypes (ALISs) in the cell lines and three ALISs in the patients. ALIS I, with high *HAT1* and low *KAT2A* expression, showed a higher sensitivity to the BETi JQ-1 than ALIS II. ALIS III had low *HAT1* expression. The TAD2B expression was low in ALIS I and II. *KAT2A* and *HAT1* expressions were negatively correlated with the methylation levels of their CG sites (*p* = 0.0004 and 0.0003). Immunological gene sets, including B cell metagenes, activated stroma-related genes, fibroblast TGF response signatures (TBRS), and T cell TBRS-related genes, were up-regulated in ALIS I. Furthermore, *KAT2A* played a key role in regulating BETi sensitivity. Conclusions: The sensitivity of ALIS I to the BETi JQ-1 may be due to the inhibition of BETi resistance pathways and genes by low *KAT2A* expression and the dysregulation of the immune microenvironment by high *HAT1* expression resulting from the absence of immune cells. ALIS I had the worst progression but showed sensitivity to BETi and B-cell-related immunotherapy, despite not responding to BRAF inhibitors.

## 1. Introduction

Melanoma is one of the most aggressive types of skin cancer. Nearly 300,000 new patients worldwide are affected by it every year. In recent years, advances in treatment, especially immune checkpoint inhibitors, have made this historically incurable cancer a potentially curable disease [1,2]. Although high cure rates have been shown with the early diagnosis and removal of melanomas, patients with stage IV metastatic disease have a five-year survival expectancy of 18% [3]. Researchers in recent decades have shown that the deregulation of the epigenome is a critical step in the activation and maintenance of aberrant transcriptional programs in melanoma pathogenesis [4,5,6,7,8].

Histone acetylation is an epigenetic post transcriptional modification that regulates gene expression and plays a crucial role in tumor development and progression [9]. In normal cells, the appropriate histone acetylation state is maintained by a combination of histone acetyltransferases (HATs) and histone deacetylases. However, this balance is often disrupted in cancer [10,11]. The bromodomain and extra-terminal (BET) proteins, which consist of BRD2, BRD3, BRD4, and BRDT (testis-specific), are “readers” of acetyl-lysine residues and recruit enzymes and transcriptional regulators to DNA regulatory elements. Previous studies have reported that BRD2 and BRD4 are overexpressed in melanoma tissues and are essential for tumor maintenance [7,12,13]. The silencing of BRD2 and BRD4, as well as treatment with BET inhibitors (BETis), can hinder the growth of melanoma.

Several specific small-molecule chemical compounds have been developed as BETis for anti-cancer functions, such as JQ-1, CPI-0610, OTX015, I-BET762, and PLX51107 [14,15,16,17,18,19]. Resistance and relapse can develop rapidly despite the efficacy of these newly targeted agents in a subset of cell lines and experimental animals [13]. Furthermore, BETis can cause various toxicities at effective doses, such as gastrointestinal toxicity, thrombocytopenia, and nausea [20,21,22]. In BETi-resistant groups, BETi treatment only increases toxicity without showing efficacy. Therefore, these groups should be excluded from treatment to improve the efficacy of BETis. Studying the mechanism of BETi resistance and identifying resistant or sensitive subtypes of melanoma are of great importance for further clinical trials and applications of BETis.

A previous study showed that the efficacy of BETis is regulated by the extent of histone acetylation [23]. *HAT1* is a classic type B histone acetyltransferase that is essential for BRD4 binding to acetylated histone H4 and initiating transcription [24,25]. The overexpression of *HAT1* promotes BRD4 binding with histone H4, resulting in the enhanced acetylation of histone H4 and elevated transcription of many genes, such as *CMYC* and *CD274* (which encodes PD-L1). *HAT1* is also involved in cancer immunity through this regulation of *PD-L1* expression [25]. The expression of *HAT1* can be decreased by ascorbate through ten-eleven translocation (TET)-mediated DNA hydroxymethylation in melanoma cell lines and mouse models. Ascorbate sensitizes melanoma cells to different kinds of BETis and enhances the efficacy of the BETi JQ-1 in treating melanoma cell lines. Moreover, ascorbate supplements sensitize melanoma xenografts to JQ-1 [23]. 

To date, a strategy to identify subtypes of cell lines and melanoma patients that are sensitive to BETis has yet to be shown. In this study, we sought to cluster melanoma cells and patients into acetylation-immune subtypes (ALISs) based on the specific roles of *HAT1* and PD-L1 in cancer treatment. The various ALISs had different acetylated and immune characteristics and may respond differently to BETis and immune therapies.

## 2. Materials and Methods

### 2.1. Analysis of Melanoma Cell Lines

**Figure 1:**
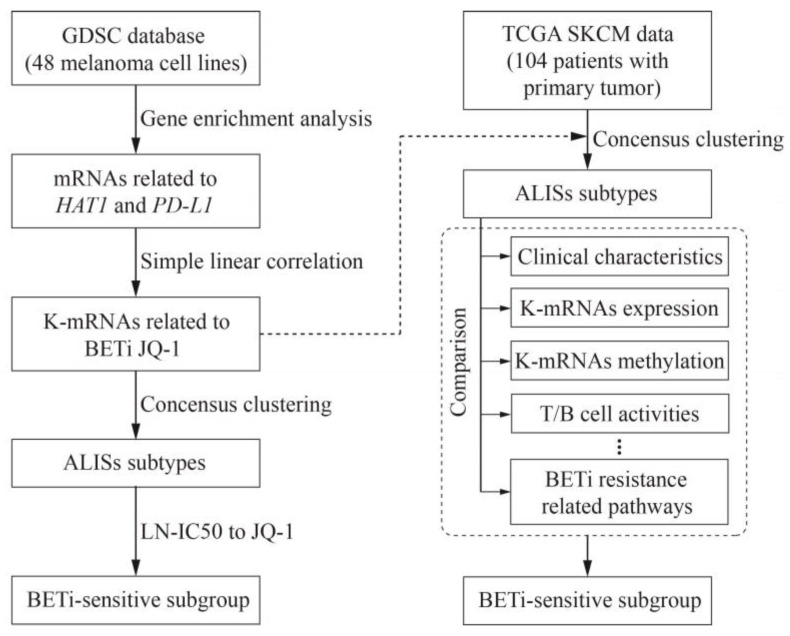
Workflow of the study design. Analyses of melanoma cell lines and TCGA SKCM datasets are performed, respectively. See detailed descriptions in the “Methods” section.

Publicly available melanoma cell line gene expression and treatment data were obtained from the Genomics of Drug Sensitivity in Cancer (GDSC) database (www.cancerRxgene.org). LN-IC50 of JQ-1 (drug ID in the database: 163) in treatment of melanoma cell lines was extracted from the GDSC database. Gene enrichment analysis (GSEA) was conducted to enrich mRNAs related to *HAT1* and PD-L1 [26,27]. Simple linear correlation analysis was performed to select K-mRNAs that were related to the sensitivity of melanoma cell lines to JQ-1 treatment. K-mRNAs were used for unsupervised clustering to identify ALISs with distinct characteristics (Figure 1).

### 2.2. Analysis of TCGA SKCM Datasets

TCGA pan-cancer data, including phenotype, gene expression RNAseq, DNA methylation and somatic mutation, were downloaded from the UCSC Cancer Browser (https://xena.ucsc.edu/welcome-to-ucsc-xena/ (accessed on 18 June 2022)). As shown in Figure 1, K-mRNAs were used for unsupervised clustering to identify ALISs in these SKCM patients. Differences in clinical characteristics, BRAF mutations, expression levels of K-mRNAs, and methylation levels of K-mRNAs were compared in different ALISs. Levels of T cell and B cell activities were also compared among different ALISs. PI networks among K-mRNAs and BETi resistance-related pathways were presented using STRING database (https://string-db.org/ (accessed on 23 June 2022)) and Cytoscape software (version 3.7.1). Genes in immunological pathways were extracted from previous published research [28,29,30].

### 2.3. Statistical Analysis

Using R package, *ConsensusClusterPlus*, unsupervised consensus clustering was performed to classify patients and cell lines into different ALISs [31]. The similarity metric was Pearson correlation with 80 percent sample resampling, 1000 iterations, up to 10 clusters, and hierarchical clustering with average inner and final linkage. A *t*-test analysis of the difference between two subtypes of LN-IC50 was performed. The overall survival (OS) of patients from different ALISs was illustrated using Kaplan–Meier survival curves, and the log rank test was used to assess survival curve disparities. Additionally, gene set variation analysis was conducted using the R *GSVA* package [32,33]. Using analysis of variation and Kruskal–Wallis test, the continuous variables of different ALISs under equal and unequal variances were compared, respectively. Multiple comparison test was based on Bonferroni method. For the test of the difference in rates, Fisher’s exact probability was calculated. Heatmaps and boxplots are presented based on R packages *pheatmap* and *ggplot2*, respectively.

Statistical analysis was performed with R platform (version 3.6.1). A two-sided *p* value less than 0.05 was considered to be statistically significant.

## 3. Results

### 3.1. Identification of Two ALISs of Melanoma Cell Lines

The gene expression data of 48 melanoma cell lines were collected from the Genomics of Drug Sensitivity in Cancer (GDSC) Project website [34]. We enriched a total of one hundred and sixty-seven mRNAs related to *HAT1* and *PD-L1*, among which nine key mRNAs (K-mRNAs, for details, see the description in the “Methods” section) were significantly correlated (using a simple linear correlation with a *p* value less than 0.05) with the natural log half-maximal inhibitory concentration (LN-IC50) of JQ-1 in the melanoma cell lines. The nine key mRNAs were *KAT2A*, *HAT1*, *ACTL6A*, *MSL1*, *MSL3*, *EP400*, *TADA2B*, *ING5,* and *HIST1H2AI*. Using the aforementioned parameters, consensus clustering was performed to obtain two to ten subtypes. Based on the consensus cumulative distribution function (CDF) plot, the cluster-consensus plot, and the item-consensus plot (Supplementary file 1: Figure S1), two distinct ALISs were successfully identified. A heatmap of the consensus matrices displayed clear boundaries between the two ALISs, indicating low confusion in the classification (Supplementary file 1: Figure S1a). Figure S1b,c show an approximate maximum for the CDF, and Figure S1d,e show a cluster consensus and an item consensus, respectively. The metrics were sufficiently large when there were two subtypes, validating the appropriateness of the two-ALIS classification. Consequently, 23 cell lines were categorized as type I (ALIS I), while 25 cell lines were classified as type II (ALIS II). Detailed descriptions of the ALIS allocation of the 48 melanoma cell lines can be found in Supplementary file 2: Table S1.

**Figure 2:**
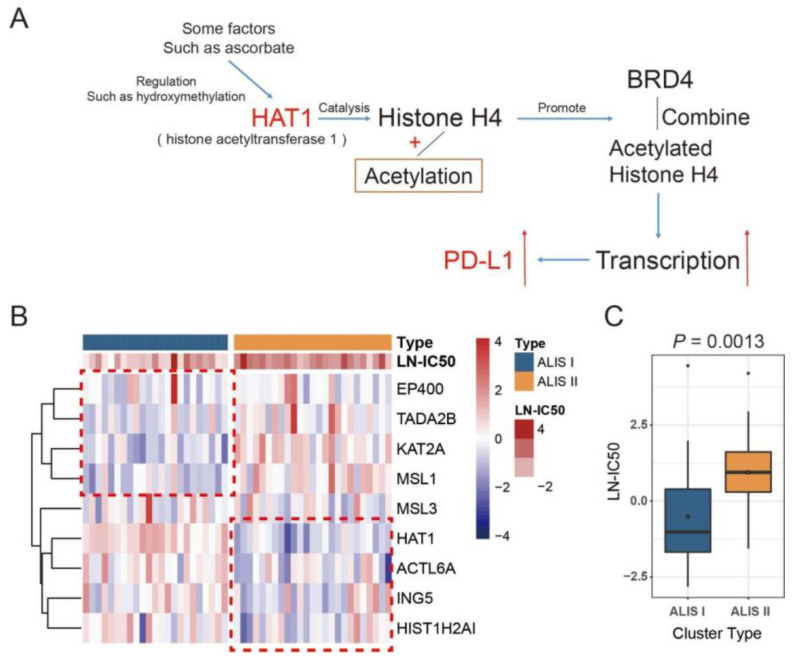
Two ALISs in melanoma cell lines. (**A**) The choice of *HAT1*- and *PD-L1*-related gene set for clustering. *HAT1* is regulated by several factors and catalyzes histone H4 acetylation. This promotes BRD4 to read the acetylated site and further initiates transcription of some genes, such as *PD-L1*. (**B**) Heatmap of nine K-mRNAs across two ALISs. Samples were sorted by ALIS. Red indicates high expression and blue indicates low expression. Regarding LN-IC50, red indicates high IC50 and blue indicates low IC50 of melanoma cell lines to JQ-1. (**C**) Boxplot of LN-IC50 in the two ALISs.

Figure 2B shows the expression of nine K-mRNAs across the two ALISs. *EP400*, *TADA2B*, *KAT2A,* and *MSL1* tended to have a lower expression in ALIS I, while *HAT1*, *ACTL6A*, *ING5,* and *HIST1H2AI* were expressed at lower levels in ALIS II. Figure 2C shows that ALIS I had a lower LN-IC50 to JQ-1 compared with ALIS II (*p* = 0.0013), which indicated that ALIS I was more sensitive to JQ-1. These results show that the nine K-mRNAs have a promising ability to discriminate JQ-1-sensitive and -resistant melanoma cell lines.

### 3.2. Identification of Three ALISs of Skin Cutaneous Melanoma (SKCM) Patients with Primary Tumors

**Figure 3:**
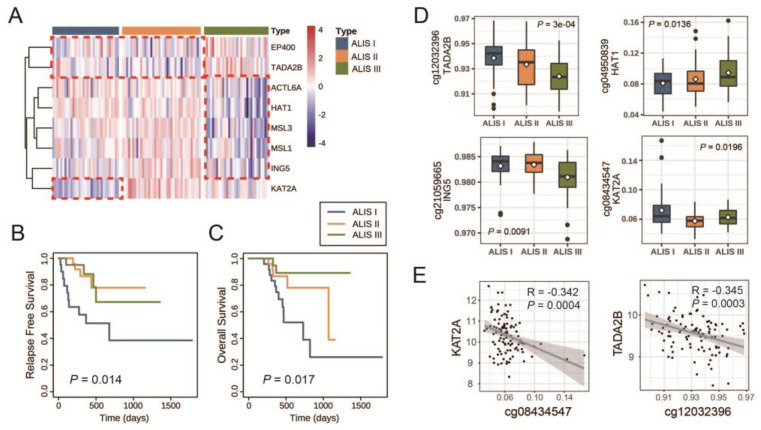
Survival characteristics and molecular characteristics in different SKCM ALISs. (**A**) Heatmap of eight K–mRNAs in three SKCM ALISs. Samples were sorted by ALISs. Red indicates high expression and blue indicates low expression. (**B**) RFS K–M curves for SKCM patients among three ALISs. (**C**) OS K–M curves for SKCM patients among three ALISs. (**D**) Boxplot of methylation levels of *TADA2B* (*p* = 3 × 10^−4^), *HAT1* (*p* = 0.0136), *ING5* (*p* = 0.0091), and *KAT2A* (*p* = 0.0196). Methylation levels are presented as β values of corresponding CG sites. (**E**) Scatterplot of levels of K-mRNAs and corresponding CG sites with statistically significant correlation coefficients.

A comprehensive analysis was conducted on a cohort of 104 SKCM patients with primary tumors, which involved the screening and integration of both clinical information and gene expression data. *HIST1H2AI* was not measured in The Cancer Genome Atlas (TCGA) database, and thus eight K-mRNAs were used for consensus clustering. Based on the CDF plot, cluster consensus plot, and item consensus plot (Supplementary file 3: Figure S2), 33 patients were classified as ALIS I, 39 as ALIS II, and 32 as ALIS II. Figure 3A presents the expression of eight K-mRNAs across the three ALISs.

### 3.3. Clinical Characteristics and Molecular Characteristics in Different SKCM ALISs

The SKCM ALISs are compared in Table 1. Sex, age, race, and BRAF mutation proportion did not differ significantly among the groups, but the tumor stage and neoplasm cancer status did. Tumors were present in 48.5% of the patients in ALIS I, significantly higher than that of the patients in ALIS II and III (*p* = 0.02). In ALIS I, there were more patients with stage IV tumors than in the other two subtypes (*p* = 0.047). 

Next, the association between the ALISs and disease progression were explored. A comparison of the survival characteristics in the SKCM ALISs is presented in Figure 3B,C and Supplementary file 3. The difference in survival is statistically significant (*p* = 0.014) (Figure 3B). In ALIS I, the median RFS was 1.9 years, and more than half of the ALIS II and III cases did not relapse until the end of their follow-up. The difference in the OS in the K–M curves across the three ALISs was also significantly different (*p* = 0.017) (Figure 3C). The median OS times of the ALIS I and II groups were 2 and 2.9 years, respectively, whereas more than half of the ALIS III patients survived until the end of their follow-up. According to these findings, the progression of the ALIS I patients was much worse than that of the ALIS II or III patients. 

A previous study demonstrated that the expression of *HAT1* was regulated through TET-mediated DNA hydroxymethylation. Therefore, we compared the methylation levels of eight K-mRNAs across the three ALISs and between any two ALISs. The results shown in Table 2 and Figure 3D demonstrated that the methylation levels of *TADA2B* (cg12032396) and *ING5* (cg21059665) were lower in ALIS III than those in ALIS I and II; the methylation level of *KAT2A* (cg08434547) in ALIS I was significantly higher than that in ALIS II, while the methylation of *HAT1* (cg04950839) in ALIS I was significantly lower than that in ALIS III. Furthermore, a simple linear correlation analysis showed a significant negative correlation between *KAT2A* and cg08434547 (R = −0.342, *p* = 0.0004) and *TADA2B* and cg12032396 (R = −0.345, *p* = 0.0003) (Figure 3E). These results demonstrated that a high expression of *TADA2B* and a low expression of *HAT1* in ALIS III and a low expression of *KAT2A* in ALIS I were likely attributable to methylated regulation, especially for *KAT2A* and *TADA2B*.

### 3.4. Immunological Characteristics across Three SKCM ALISs

**Figure 4:**
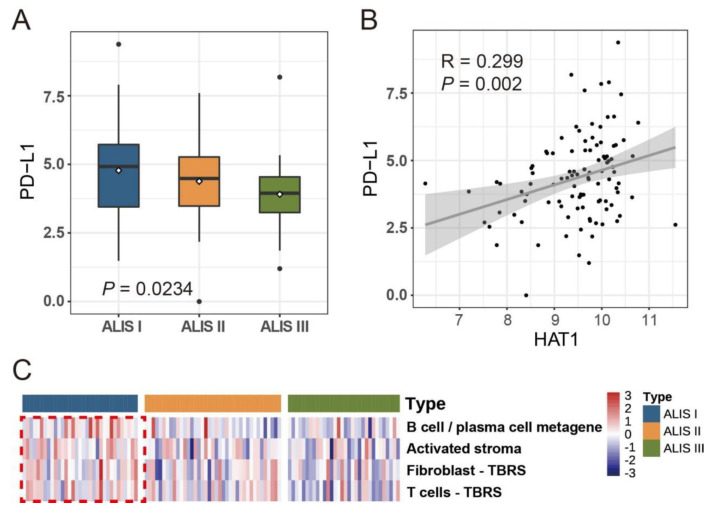
Immunological characteristics across three SKCM ALISs. (**A**) Boxplot of *PD-L1* mRNA expression level among three ALISs. (**B**) Scatterplot of *PD-L1* and *HAT1*. (**C**) Heatmap of immunological pathways among different ALISs.

Previous studies indicate that *HAT1* regulates cancer immunity. Therefore, we further studied the immunological characteristics across the three ALISs. Figure 4A showed that the expression of *PD-L1* was statistically different among the three ALISs (*p* = 0.0234); moreover, the expression of *PD-L1* in ALIS III was lower than that in ALIS I, although the difference was not significant (*p* = 0.072). As shown in Figure 4B, there was a significant positive correlation between *HAT1* and *PD-L1* (R = 0.299, *p* = 0.002). Regarding immunological gene sets, four cancer-related immunological gene sets were analyzed across the three ALISs: the B cell/plasma cell metagene, the activated stroma-related gene set, the fibroblast TGF β response signature (TBRS)-related gene set, and the T cell TBRS-related gene set. Figure 4C demonstrates that these four immunological gene sets were all significantly expressed at high levels in ALIS I.

### 3.5. Protein Interaction (PI) Network Reveals the Vital Role of KAT2A in Regulating BETi Resistance-Related Biological Genes and Pathways

**Figure 5:**
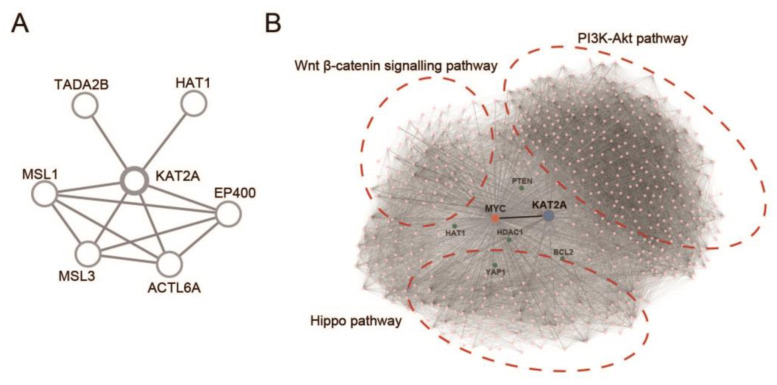
Protein interaction network. (**A**) Protein interaction network between eight K–mRNAs used for unsupervised clustering in SKCM patients. (**B**) Protein interaction network between *KAT2A*, MYC, and several BETi resistance-related pathways.

Next, we constructed a PI network between eight K-mRNAs. As shown in Figure 5a, *KAT2A* plays a vital and core role with the highest degree in the protein regulation network. Figure 5b shows that *KAT2A* had a PI regulation relationship with BETi resistance-related biological genes and pathways either directly or through MYC. These results demonstrate that *KAT2A* is not only a vital factor in histone acetylation, but also a core component in the regulation of BETi resistance.

## 4. Discussion

Metastatic melanoma remains a mostly incurable disease despite the high cure rates that are associated with the early diagnosis and removal of melanomas. To date, immune therapies are considered to be a preferred treatment strategy for melanoma, given that melanoma is regarded as an immunogenic tumor. As a first-line treatment in metastatic melanoma, anti-CTLA-4 and anti-PD-1 have improved the OS rate of many patients [35]; however, the majority of patients treated with monotherapy are non-responsive [36], and combinations of anti-PD-1 with anti-CTLA-4 lead to grade III–V adverse events [37]. Hence, physicians may also choose targeted therapy in the initial treatment according to the clinical situation of their patients. 

Approximately 40% to 60% of malignant melanoma cases carry mutations in BRAF, which is the most frequent mutation event in melanoma [38,39]. Current targeted therapy agents for melanoma aim to block BRAF and MEK proteins. These agents show marked antitumor effects against melanoma cell lines with BRAF mutations but no effects against cells with wild-type BRAF [40,41,42]. ALIS I patients showed the worst progression but no differences in BRAF mutation frequency compared with that of the other subtypes. Thus, these patients might not benefit from BRAF inhibitors.

There has been growing interest in epigenetic regulators over the past several years of cancer research [43,44]. As epigenetic “reader” domains, BET family proteins bind to acetylated lysines on histone tails, resulting in the formation of transcriptional complexes that can drive the expression of a number of target genes involved in cancer progression [45,46,47,48,49]. BETis have been reported to have significant antitumor activities in melanoma [50] not only through inhibiting the transcription of oncogenes but also by impacting pro- and anti-inflammatory responses. In the current report, by clustering melanomas using histone acetylation- and immunotherapy-related genes, we identified subtypes with different responses to BETis and immunotherapy.

In this study, melanoma cell lines were clustered into two ALISs based on nine K-mRNAs, and ALIS I showed a greater sensitivity to the BETi JQ-1 compared with that of ALIS II. Furthermore, SKCM patients were clustered into three ALISs based on eight K-mRNAs, and ALIS I showed the worst progression. A previous study showed that *HAT1* enhances the efficacy of JQ-1 in treating melanoma cell lines [23]; however, *HAT1* was expressed at a high level in ALIS I (Figure 2B). This suggests that other molecular features play a vital role in regulating the sensitivity of melanoma to JQ-1. *KAT2A* is a *HAT* that is expressed at low levels in the ALIS I cell line and ALIS I SKCM patients. Therefore, we hypothesized that *KAT2A* might be another vital factor in regulating BETi resistance. Figure 5 shows that *KAT2A* plays a crucial role not only among the eight K-mRNA regulated pathways, but also in the regulation of BETi sensitivity-related proteins and pathways. These proteins and pathways, including BCL2, YAP1, PTEN, HDAC1, the PI3k-Akt-mTOR pathway, and the Hippo pathway [15,51,52,53], were either regulated directly or through MYC by *KAT2A* (Figure 5b). These results suggest that the inhibition of *KAT2A* could downregulate BETi resistance-related pathways and further sensitize melanomas to BETis. This contribution could complement those of previous studies [54,55,56,57].

**Figure 6:**
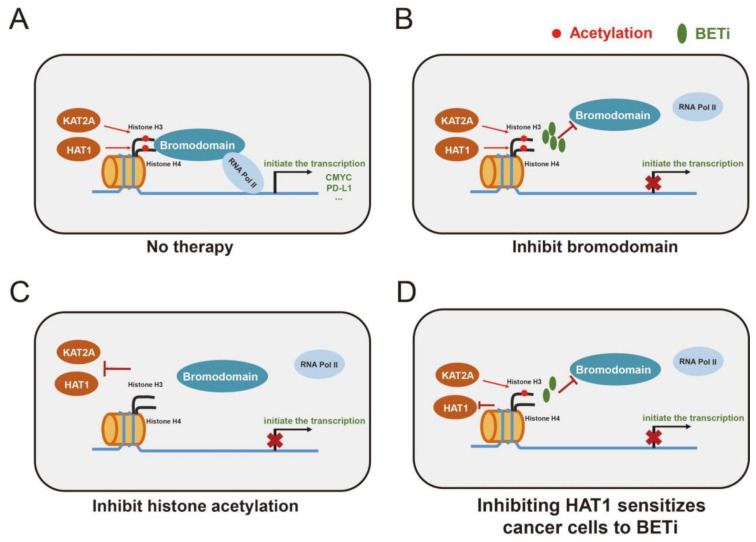
The mechanism of BETis and histone acetylation inhibitors in cancer. (**A**) Identification of histone acetylation by bromodomain and initiation of transcription of genes, including *CMYC* and *CD274* (which encodes PD-L1). (**B**) BETi inhibits the identification of histone acetylation by bromodomain, reduces the recruitment of RNA polymerases II, and downregulates transcription of downstream mRNAs. (**C**) Inhibition of *KAT2A* and *HAT1* inhibits acetylation of histone H3 and H4 and downregulates transcription of downstream mRNAs. (**D**) Inhibition of *HAT1* inhibits acetylation of histone H4 and sensitizes cancer cells to BETi.

*HAT1* also functions as a transcription factor to regulate the tumor microenvironment, sensitivity to immunotherapy, and the expression of various genes, such as *BCL2L12* [58] and *FAS* [59]. Figure 6 presents an overview of the mechanisms of BETis and histone acetylation inhibitors in cancer. Regarding immunological pathways, Figure 4C shows that the four selected cancer-related biological gene sets were all overexpressed in the ALIS I SKCM patients. B cell/plasma cell immune genes were significantly and positively associated with a favorable chemotherapy response in patients with breast cancer. The anti-tumor functions of plasma B cells evoked a response to neoadjuvant chemotherapy [30]. Activated stroma as well as T cell TBRS and fibroblast TBRS pathways were positively associated with poor progression in pancreatic ductal adenocarcinoma and colorectal cancer [30].

Mustafi et al. reported that *HAT1* could be regulated through methylation and demethylation [23], and thus we studied the correlation of mRNA expression to the methylation levels of K-mRNAs. Figure 3D shows that the high expression of *TADA2B* in ALIS III, the low expression of *HAT1* in ALIS III, and the low expression of *KAT2A* in ALIS I could possibly be attributed to hyper- or hypomethylation. Figure 3E indicates that *KAT2A* and *TADA2B* were more likely to be regulated through methylation.

Based on the above results, we hypothesize that the low expression of *KAT2A* in ALIS I melanoma cell lines inhibits pathways related to JQ-1 resistance, while high *HAT1* expression was unable to regulate the immune microenvironment because there were no immune cells in the culture bottles, which led to the ALIS I cell line’s sensitivity to JQ-1. In ALIS I SKCM patients, poor progression can be attributed to the overexpression of activated stroma-related genes, T cells TBRS, fibroblast TBRS, and *PD-L1* rather than BRAF mutations. However, the low expression of *KAT2A* in ALIS I patients suggests that these patients may be sensitive to BETis and histone acetylation inhibitors despite the fact that these patients suffered the worst progression and had no response to inhibitors of mutated BRAF. The activated B cell/plasma cell immune genes in ALIS I indicate that these cases may have responses to treatment related B cell immunity.

Furthermore, the findings of our study have potential clinical implications. For instance, by demonstrating that the inhibition of *KAT2A* downregulates BETi resistance-related pathways and sensitizes melanoma to BETis, our research suggests a potential strategy to enhance the effectiveness of BETi treatment in melanoma patients. This information could contribute to the development of more targeted and personalized therapeutic approaches for melanoma, ultimately improving patient outcomes. Further clinical studies and validation are still needed to fully assess the clinical relevance and potential translation of our findings into patient care.

To date, limited information is available on BETi therapy in cancer patients. Over 20 clinical trials on BETi are ongoing according to the ClinicalTrials.gov database (https://clinicaltrials.gov/ct2/home (accessed on 1 July 2022)). Here, we analyzed two public datasets and identified ALISs in melanoma cell lines and SKCM patients with different molecular characteristics. Our results have provided information for further research on clinical treatment and BETi-resistance/sensitivity mechanisms. However, this study has certain limitations. First, JQ-1 is only one BETi, and more studies are needed on the mechanisms of resistance to other BETi, such as OTX-015, I-BET-762, and CPI-0610. Second, the entire study is in silico with published datasets. This study has not formally demonstrated whether ALIS I SKCM patients are BETi-sensitive and immunotherapy-sensitive. However, the current study is limited to the data of melanoma patients treated with BETis. Through our analysis of multiomics data and an extensive literature review, we have provided important findings that could be the basis of further research on BETis in melanoma.

## 5. Conclusions

We performed a multiomics data analysis of 48 melanoma cell lines and 104 SKCM patients. A novel acetylation-immune subtype of melanoma was proposed. Using this subtyping strategy, a BET-inhibitor-sensitive subgroup was identified. It suggested a potential way to enhance the effectiveness of BETi treatment in melanoma patients. Specifically, the ALIS I cell lines were sensitive to JQ-1 compared with the ALIS II cell lines, and this sensitivity was considered to be attributable to the inhibition of BETi resistance-related pathways and genes by the low expression of *KAT2A* and the loss of the ability to regulate the immune microenvironment by the high expression of *HAT1* due to the lack of immune cells. The ALIS I SKCM patients suffered the worst progression, yet they were considered to be sensitive to BETi and B-cell-related immunotherapy, though they had no response to inhibitors of mutated BRAF.

## Figures and Tables

**Table 1:** Baseline characteristics of the three identified ALISs.

Characteristics ^a^	ALIS I (N = 33)	ALIS II (N = 39)	ALIS III (N = 32)	*p* Value
Sex (N, %)				0.659
Male	17 (58.6)	20 (64.5)	12 (52.2)	
Female	12 (41.4)	11 (35.5)	11 (47.8)	
total	29	31	23	
Age ($\bar{x}\pm s$)	63.52 ± 15.05	62.68 ± 14.63	63.91 ± 13.31	0.939
AJCC Stage (N, %) ^b^				**0.047**
Stage I	0 (0)	3 (8.1)	0 (0)	
Stage II	23 (69.7)	26 (70.3)	18 (60)	
Stage III	7 (21.2)	8 (21.6)	12 (40)	
Stage IV	3 (9.1)	0 (0)	0 (0)	
total	33	37	30	
Neoplasm Cancer Status (N, %)				**0.020**
Tumor-free	17 (51.5)	30 (76.9)	28 (87.5)	
With tumor	16 (48.5)	9 (23.1)	4 (12.5)	
total	33	39	32	
Race (N, %)				0.888
Asian	3 (9.1)	2 (5.3)	2 (6.5)	
White	30 (90.9)	36 (94.7)	29 (93.5)	
total	33	38	31	
BRAF mutation (N, %)				0.558
Wild	12 (42.9)	9 (34.6)	11 (50)	
Mutated	16 (57.1)	17 (65.4)	11 (50)	
total	28	26	22	

^a^ Column percentages of categorical characteristics are presented. ^b^ AJCC Stage: the eighth edition of American Joint Cancer Committee Stage.

**Table 2:** Comparison of methylation levels of eight K-mRNAs among three ALISs ^a^.

CG Sites	Gene Symbol	*p* Value	I vs. II ^b^	I vs. III ^b^	II vs. III ^b^
cg12032396	TADA2B	**3 × 10^−4^**	0.5050	**0.0008**	0.0358
cg04950839	HAT1	**0.0136**	0.9110	**0.0420**	0.3520
cg21059665	ING5	**0.0091**	1.0000	**0.0210**	**0.0250**
cg08434547	KAT2A	**0.0196**	**0.0160**	0.8280	0.3520
cg24545687	EP400	**0.0351**	1.0000	0.1100	0.4300
cg25198545	ACTL6A	0.8958	-	-	-
cg09665160	MSL1	0.9840	-	-	-
cg20205061	MSL3	0.2193	-	-	-

^a^ *p* value adjustment method: Bonferroni. ^b^ *p* value of the comparison between any two ALISs.

## Data Availability

Data in this study were generated by the TCGA Research Network: http://cancergenome.nih.gov/ (accessed on 23 June 2022) and GDSC database: https://www.cancerrxgene.org/ (accessed on 23 June 2022).

## Supplementary Materials

The following supporting information can be downloaded at: https://www.mdpi.com/article/10.3390/ph16071037/s1, Figure S1: Results of unsupervised consensus clustering in the GDSC dataset; Table S1: ALISs of 48 melanoma cell lines; Figure S2: Results of unsupervised consensus clustering in TCGA SKCM patients with a primary tumor; Table S2: Comparison of immunological pathways among three ALISs.

## Abbreviations

HAT: histone acetyltransferases; BET: bromodomain and extra-terminal; BETi: BET inhibitor; TET: ten-eleven translocation; ALIS: Acetylation-Immune Subtype; RFS: relapse free survival; OS: overall survival; GDSC: Genomics of Drug Sensitivity in Cancer; K-mRNAs: key mRNAs; LN-IC50: natural log half maximal inhibitory concentration; CDF: cumulative distribution functions; SKCM: skin cutaneous melanoma; TCGA: The Cancer Genome Atlas; K–M: Kaplan–Meier; TBRS: TGF-β response signatures; PI: protein interaction; GSEA: gene enrichment analysis.
