# Supplementary material for: A Novel Acetylation-Immune Subtyping for the Identification of a BET Inhibitor-Sensitive Subgroup in Melanoma
Source: Pharmaceuticals (Basel). 2023 Jul 21;16(7):1037. doi: 10.3390/ph16071037 (PMC10383501; doi:10.3390/ph16071037)
Supplement: Supplementary file 1 [file pharmaceuticals-16-01037-s001.zip › Table S1.docx]

**Table S1.** ALISs of 48 melanoma cell lines.

| Cell line name | ALIS | Cell line name | ALIS | Cell line name | ALIS | Cell line name | ALIS |
| --- | --- | --- | --- | --- | --- | --- | --- |
| 451Lu | I | MZ7-mel | I | C32 | II | LB2518-MEL | II |
| A101D | I | SH-4 | I | COLO-783 | II | LB373-MEL-D | II |
| A2058 | I | SK-MEL-1 | I | COLO-792 | II | LOXIMVI | II |
| CHL-1 | I | SK-MEL-24 | I | COLO-800 | II | MEL-JUSO | II |
| COLO-679 | I | SK-MEL-28 | I | COLO-829 | II | Mewo | II |
| CP50-MEL-B | I | SK-MEL-3 | I | CP66-MEL | II | RPMI-7951 | II |
| G-361 | I | SK-MEL-5 | I | GAK | II | RVH-421 | II |
| IGR-1 | I | UACC-62 | I | G-MEL | II | SK-MEL-2 | II |
| IPC-298 | I | WM1552C | I | HMV-II | II | SK-MEL-30 | II |
| IST-MEL1 | I | WM35 | I | HT-144 | II | UACC-257 | II |
| M14 | I | WM793B | I | IGR-37 | II | WM-115 | II |
| MZ2-MEL | I | A375 | II | K2 | II | WM278 | II |
