# Supplementary material for: A Novel Acetylation-Immune Subtyping for the Identification of a BET Inhibitor-Sensitive Subgroup in Melanoma
Source: Pharmaceuticals (Basel). 2023 Jul 21;16(7):1037. doi: 10.3390/ph16071037 (PMC10383501; doi:10.3390/ph16071037)
Supplement: Supplementary file 1 [file pharmaceuticals-16-01037-s001.zip › Table S2.docx]

**Table S2.** Comparison of immunological gene sets among three ALISs.

| Pathway | *P* value | Gene Symbols ^a^ | Entrez Gene ID |
| --- | --- | --- | --- |
| B cells / plasma cell metagenes | 0.004 | TNFRSF17, CD79A, IGHA1, IGHA2, IGHD, IGHG1, IGHG2, IGHG3, IGHG4, IGHM, IGJ, IGKC, IGL, IRF4, NTN3, POU2AF1, FAM20B, CKAP2, IGHV4-31, IGHV3-23, IGHV1-69, IGLV1-44, IGLJ3, IGKV1D-13, IGKV4-1, IGKV3-20, IGK, LAX1, GUSBP11, IGLL3 , IGKV1OR10-1, IGH | 608, 973, 3493, 3494, 3495, 3500, 3501, 3502, 3503, 3507, 3512, 3514, 100423062, 3662, 4917, 5450, 9917, 26586, 28396, 28442, 28461, 28823, 28831, 28902, 28908, 28912, 50802, 54900, 91316, 91353, 642424, 102723407 |
| Activated stroma | 0.0181 | SPARC, COL1A2, COL3A1, POSTN, COL5A2, COL1A1, THBS2, FN1, COL10A1, COL5A1, SFRP2, CDH11, CTHRC1, FNDC1, SULF1, FAP, LUM, COL11A1, ITGA11, MMP11, INHBA, VCAN, GREM1, COMP | 6678, 1278, 1281, 10631, 1290, 1277, 7058, 2335, 1300, 1289, 6423, 1009, 115908, 84624, 23213, 2191, 4060, 1301, 22801, 4320, 3624, 1462, 26585, 1311 |
| Fibroblast - TBRS | 0.0314 | FLT1, COL10A1, IGFBP3, NOX4, MEX3B, GAS1, INHBA, VEGFA, CDKN2B, FBXO32, CALB2, CTGF, KANK4, NET1, HEY1, SERPINE1, ESM1, TIMP3, SYNE1, BHLHE40, PLAUR, APBB2, FGF1, ANGPTL4, LMCD1, PGM2L1, KAL1, TNFAIP6, OSGIN2, PODXL, PCDH9, C13orf33, RASGRP3, LOH3CR2A, SPSB1, FN1, GADD45B, TRIB1, STK17B, KLF7, LRRC8C, FNIP2, TGFB2, FRMD4A, CNTN1, NGF, NUAK1, EFNB2, TSPAN2, CHST11, EGR2, DAAM1, PALLD, GPR161, CALD1, LOC728449, HIVEP2, RHOU, KDM6B, FOXP1, ELMOD1, SEMA7A, PTHLH, PMEPA1, HAS2, SNORD114-3, GRB14, LIF, TSHZ3, PDLIM4, LOC728264, ZNF365, PDGFC, JUNB, CILP, BPGM, ARHGEF3, PGBD5, TAGLN3, TUFT1, GPR183, S1PR5, CLDN4, MBOAT2, CNNM4, DNAJB5, C3orf52, DHRS2, SOX4, EPHA4, COL27A1, SMAD7, F2RL1, LOC100128178, RASL12, SLC35F2, SETBP1, DOCK10, C5orf13, DNAJC18, DACT1, WNT9A, ETV6, FGF18, HBEGF, TNC, SDC1, KIAA1755, EDN1, ITGB6, PTGS2, PLEK2, LOC201651, STEAP2, SLC46A3, SNX30, RYBP, TMEM49, SORBS2, HIC1, NEDD9, ARHGEF40, IFIH1, GZMK, VEPH1, PIK3CD, IL6, YIPF5, SKIL, RASD1, JARID2, IL11, SNAI1, SOX6, STK38L, NKX3-1, CDH6, PELI1, PRDM1, PDPN, WNT2, LMO4, C4orf26, CACHD1, PRR5L, TMEM2, DDX10, MTSS1, CLDN14, JHDM1D, SLC19A2, PLCE1, PRR9, MEGF9, GOPC, MSC, PPP1R14C, PKNOX2, MSX2, SNCAIP, SLC35F3, LOC727930, HS3ST3B1, MEOX1, E2F7, AUTS2, FUT4, DLX2, TBX3 | 2321, 1300, 3486, 50507, 84206, 2619, 3624, 7422, 1030, 114907, 794, 1490, 163782, 10276, 23462, 5054, 11082, 7078, 23345, 8553, 5329, 323, 2246, 51129, 29995, 283209, 3730, 7130, 734, 5420, 5101, 84935, 25780, 29931, 80176, 2335, 4616, 10221, 9262, 8609, 84230, 57600, 7042, 55691, 1272, 4803, 9891, 1948, 10100, 50515, 1959, 23002, 23022, 23432, 800, 728113, 3097, 58480, 23135, 27086, 55531, 8482, 5744, 56937, 3037, 767579, 2888, 3976, 57616, 8572, 728264, 22891, 56034, 3726, 8483, 669, 50650, 79605, 29114, 7286, 1880, 53637, 1364, 129642, 26504, 25822, 79669, 10202, 6659, 2043, 85301, 4092, 2150, 100628315, 51285, 54733, 26040, 55619, 9315, 202052, 51339, 7483, 2120, 8817, 1839, 3371, 6382, 85449, 1906, 3694, 5743, 26499, 201651, 261729, 283537, 401548, 23429, 81671, 8470, 3090, 4739, 55701, 64135, 3003, 79674, 5293, 3569, 81555, 6498, 51655, 3720, 3589, 6615, 55553, 23012, 4824, 1004, 57162, 639, 10630, 7472, 8543, 152816, 57685, 79899, 23670, 1662, 9788, 23562, 80853, 10560, 51196, 574414, 1955, 57120, 9242, 81706, 63876, 4488, 9627, 148641, 727930, 9953, 4222, 144455, 26053, 2526, 1746, 6926 |
| T cells - TBRS | 0.0062 | TIMP1, RAB31, CLIC4, MXRA7, SERPINE1, INPP5F, GEM, ANXA5, LMCD1, CST6, RBPJ, RASGRP3, PLAU, LOH3CR2A, ITGAV, MAP4, KLF7, ABHD2, BMP1, KCNK1, RGS16, ATXN1, NMB, EGR2, IL1RN, KLF10, APOD, PIK3IP1, CRIP1, SLC5A3, NR4A3, EVI2A, FAM102A, TIAM1, ALOX5AP, ZNF365, TNFSF4, PPAP2A, PKIA, FBXO11, DCLRE1C, SOX4, C18orf1, NPTX1, CSGALNACT1, TMOD1, ATP1B1, CCR4, CD83, LRIG1, ADO, LRBA, DIXDC1, SYNJ2, RIMS3, JUN, CD96, PRKCD, S100PBP, HLF, ABCC4, CXCL13, PPARG, AHCYL2, AQP3, TSPAN13, SLC1A4, TFEB, ITGAE, APOBEC3G, CDYL, IL7R, IL9, ZNF238, MYH6, GPR35, MB | 7076, 11031, 25932, 439921, 5054, 22876, 2669, 308, 29995, 1474, 3516, 25780, 5328, 29931, 3685, 4134, 8609, 11057, 649, 3775, 6004, 6310, 4828, 1959, 3557, 7071, 347, 113791, 1396, 6526, 8013, 2123, 399665, 7074, 241, 22891, 7292, 8611, 5569, 80204, 64421, 6659, 753, 4884, 55790, 7111, 481, 1233, 9308, 26018, 84890, 987, 85458, 8871, 9783, 3725, 10225, 5580, 64766, 3131, 10257, 10563, 5468, 23382, 360, 27075, 6509, 7942, 3682, 60489, 9425, 3575, 3578, 10472, 4624, 2859, 4151 |
